# A Structural Model for Aggression in Middle School Students in Korea: Based on Ajzen’s Theory of Planned Behavior

**DOI:** 10.3390/ijerph20021576

**Published:** 2023-01-15

**Authors:** Sook Jang, Hye Young Ahn

**Affiliations:** 1 Department of Nursing, Honam University, Gwangju Metropolitan City 62399, Republic of Korea; 2 College of Nursing, Eulji University, Uijeongbu 11759, Republic of Korea

**Keywords:** middle school students, aggression, structural model, parenting behavior, aggression regulation intention, Theory of Planned Behavior

## Abstract

(1) Purpose: The purpose of this study was to construct a hypothetical model for the variables that can explain aggression in middle school students in order to promote mental health and the growth and development of middle school students in Korea. Through this model, we tried to confirm the structural relationship between the variables and their influence. (2) Methods: The subjects of this study were middle school students in the Korean city of D, and the data collection period was from July to September 2016. The data were collected from the final 310 completed copies of the questionnaire, excluding 23 copies with insufficient data. SPSS 26.0 and AMOS 26.0 were used for data analysis. (3) Results: The fit indices of the final model (GFI = 0.88, AGFI = 0.84, IFI = 0.95, CFI = 0.95, SRMR = 0.06, and RMSEA = 0.07) met the acceptable levels. The variables that affected middle school students’ aggression were parenting behavior and aggression regulation intention, and together, their explanatory power for aggression was 50.5%. Perceived behavioral control was the most influential variable for aggression regulation intention. (4) Conclusions: The results of this study will be used as a basis for studying aggression and developing aggression control training programs with the aim of reducing aggression in middle school students.

## 1. Introduction

### 1.1. The Necessity of Research

Early adolescence stage is a period in which balanced growth and development and the identity of a mature personality should be established. Compared with rapid physical growth, emotional and psychological development are immature. In the process of coping with these issues, adolescents feel conflict and confusion, and experience stress and aggression [1]. Aggression is an intentional behavior motivated by causing harm to others. In addition, it can be an accidental behavior that can occur without intentional behavior [2]. Aggression in early adolescence manifests itself in immature ways of harming others and expressing outright anger [3].

Aggression expressed in the adolescent stage continuously increases and causes aggressive behavior that leads to drug use, delinquency, and chronic violence, even in adulthood [4]. In addition, since aggression at a middle school age is a major variable that predicts social success, criminal behavior, and psychological–social function, it affects human growth and development [5]. The problem behavior experienced in the adolescence stage is delinquency, and among the individual factors related to adolescents’ delinquency, aggression is a representative cause [6]. Aggression not only has a positive effect on delinquency, but a high aggression tendency in middle school students increases the level of delinquency in high school students [7]. Therefore, for desirable adolescent growth and development, preventive interventions for aggression in adolescents are needed as soon as possible. 

Aggression is manifested as the interaction between an individual’s internal and external factors [8]. The important internal factors of aggression include self-esteem, impulsivity, anxiety, depression, and emotion regulation [9,10]. External factors include daily stress, parenting behavior, social support, etc. [1,11,12]. In particular, the biggest variable affecting adolescents’ problem behavior was parenting behavior [13]. Parenting behavior was closely related to adolescent aggression. 

Moreover, aggression is closely related to emotion regulation. Emotion regulation refers to an awareness and understanding of emotions, acceptance of emotions, regulation of impulsive behavior when experiencing negative emotions, the ability to behave in harmony with individual goals, and the ability to use emotion regulation strategies appropriately for the situation [13,14]. That is, when a person feels confused, if they can manage their emotions well and calm themselves down effectively, they will not feel confused in situations that induce aggressive behavior. However, the lower the ability to regulate emotion, the more likely a person is to show aggressive behavior [15,16]. 

In the meantime, there have been many studies on the factors that affect aggression in adolescents [1,11,12,13,17]. At the same time, they were studies on the relationship between factors influencing aggression [9,10]. However, there were few studies that suggest a systematic theoretical model to understand adolescents’ aggression. Recently, a study on the prediction of aggression in male adolescents using the Theory of Planned Behavior (TPB) was conducted [18], but no additional variables were added to increase the explanatory power of the study. The TPB should make efforts to introduce additional variables to increase the explanatory power of behavior [19]. Therefore, through a literature review, this study found that the variables most related to aggression were parenting behavior and emotion regulation variables and added these two variables to the TPB.

According to the TPB, intention is a decisive factor for behavior, and intention explains behavior [19]. The TPB, applied to aggression, indicates that attitudes toward aggression regulation behavior, subjective norms, and perceived behavioral control affect aggression regulation intention, and aggression regulation intention has a decisive effect on aggression [20]. 

Therefore, based on Ajzen’s TPB [19], which can explain aggression and considers all psychosocial factors, this study attempted to develop an integrated model that could systematically and comprehensively explain aggression in middle school students. Furthermore, for the sound development of mental health in middle school students, this study attempted to understand the factors affecting aggression. In addition, it is intended to be used as basic data for developing a nursing intervention program for middle school students to manage aggression and solve school violence problems.

### 1.2. Research Purpose

Based on Ajzen’s TPB [19], the purpose of this study was to construct a model to explain the aggression of middle school students by selecting the variables affecting aggression and to promote the proper mental health development of middle school students. Additionally, the study analyzed how parenting behavior and emotion regulation affect aggression, and verified the structural relationships among the factors. The specific aims are as follows: (1) identify factors affecting middle school students’ aggression. (2) Establish a hypothetical model for middle school students’ aggression. (3) Verify the fit between the hypothetical model and actual data. (4) Present a model explaining middle school students’ aggression.

### 1.3. Conceptual Framework

The TPB explains attitudes toward behavior, subjective norms, and behavior in relation to these norms, and adds a variable called perceived behavioral control. According to the TPB, intention is a decisive factor for behavior. Aggression is the intention to achieve one’s goals in a conflict situation, and it is a behavior that uses physical or verbal force [21]. As a result, the intent to attack indicates a behavior called aggression, and the intention within the TPB is consistent with the conceptual framework that explains the behavior.

The TPB was applied as a health belief model and as a theory that explains health behavior [22,23,24]. Recently, it has been applied to identify the determinants of various deviant behaviors such as illegal downloading and uploading of digital content, cyber violence, and prostitution tourism intentions, and to establish appropriate response strategies [25,26,27]. In addition, it was applied as a theoretical framework in structural model studies to analyze, for example, aggression in university athletes and the spiritual nursing performance of clinical nurses. The empirical theory was also verified [28,29]. The TPB suggests that efforts should be made to include additional variables to increase the explanatory power regarding behavior [30].

**Figure 1:**
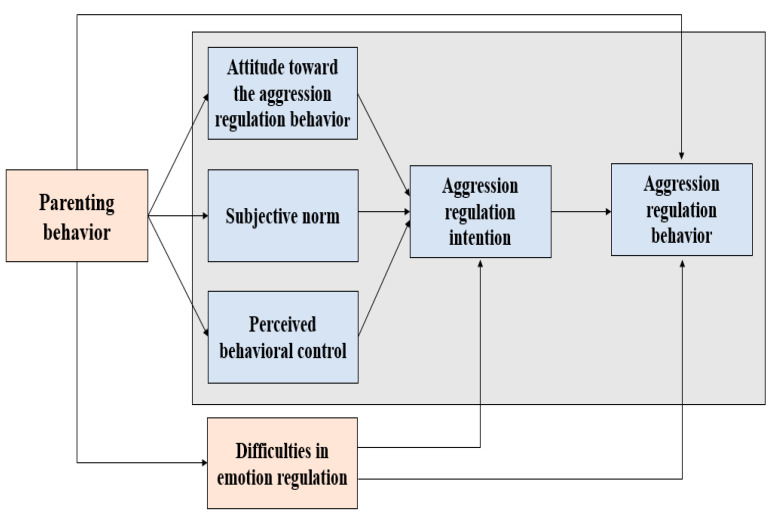
Conceptual framework.

In addition, failure to regulate emotion has important effects on adolescents’ anger and aggressive behavior, dissatisfaction, and eating disorders [31]. Conversely, it has been reported that when the ability to regulate emotion is healthy, the likelihood of expressing aggressive or problematic behavior is low, mental health is good, and psychological wellbeing is higher [32,33]. Therefore, in this study, difficulties in emotion regulation were set as an endogenous variable. As it is desirable to measure positive aggression regulation behavior rather than measuring aggression, which is a negative emotion, and because the specific subjects of the study were middle school students [34], in this study, attitudes toward aggression regulation behavior, subjective norms for aggression regulation behavior, perceived behavioral control in terms of aggression regulation behavior, and aggression regulation intention were set as the variables of the TPB. Parenting behavior affects attitudes toward aggression regulation behavior, subjective norms, perceived behavioral control, and aggression, whereas emotion regulation has been reported as a variable affecting intention [35,36,37,38,39]. Therefore, in this study, a conceptual framework was constructed by considering that the application of the TPB with regard to aggression is influenced by parenting behavior and affects middle school students’ aggression alongside difficulties in emotion regulation (Figure 1).

## 2. Materials and Methods

### 2.1. Research Design

In this study, a hypothetical model was constructed to explain middle school students’ aggression based on a literature review [11,12,13,14,15,17] and the TPB [19]. This is a cross-sectional structural model validation study to verify the fit and hypotheses of the model regarding the TPB variables, parenting behavior, difficulties in emotion regulation, aggression regulation intention, and aggression.

### 2.2. Research Participants

The subjects of this study were middle school students from two schools residing in city D. The specific criteria for the selection of subjects were male and female middle school students who understood the purpose of this study and agreed to participate, did not have developmental disabilities, and were able to communicate. It is recommended that the minimum sample size of the structural equation model be 15 per measurement variable. Moreover, if the sample size is small, the problem of representativeness is raised for all parameters in the sample; thus, it is preferable to use 200 or more samples as a reference [40]. Therefore, considering the measurement variables of this research analysis and the dropout rate of incomplete questionnaires, 340 questionnaires were distributed in total, and 333 copies were collected to secure a sufficient number of samples for the study. Of these, 310 copies (93.1%) were used for the final analysis, excluding 23 copies (6.9%) showing data with insincere answers or missing data.

### 2.3. Research Measurement Tools

#### 2.3.1. Parenting Behavior Perceived by Children

On the basis of Baumrind’s theory [41], and according to the parenting behavior scale that affects psychosocial and social maladjustment constructed by Park and Kim [42], and the scale developed by Barber [43] to measure the psychological control of children, this study used a parenting behavior scale that Kim [44] reconstructed by adding sections on intimacy, autonomy, and control of early adolescents. The tool contains four subtypes (intimacy and rationality, control, overprotection, and neglect), and each subtype contributes a total of 38 items: 19 items, 11 items, 4 items, and 4 items, respectively. In this study, parenting behavior was divided into two factors, positive parenting behavior (intimacy and rationality) and negative parenting behavior (control, overprotection, and neglect), which was constructed and measured. If we consider the concept of these two factors, for positive parenting behavior, the higher the score, the greater the positive parenting behavior. This means that the parenting behaviors of intimacy and rationality increase. Conversely, for negative parenting behavior, the higher the score, the greater the negative parenting behavior. In other words, this means that the parenting behaviors of control, overprotection, and neglect are high. However, since two measurement variables in this case have opposite directions within one latent variable, the negative parenting behavior was measured by inverse conversion in order to align the two factors in the same direction [40]. Therefore, to reinterpret the parenting behavior in this study, it was determined that the higher the parenting behavior score, the higher the positive parenting behavior and the lower the negative parenting behavior. The item format is a 5-point Likert scale (1 = strongly disagree, 2 = somewhat disagree, 3 = moderate, 4 = somewhat agree, and 5 = strongly agree), and the total score ranged from 38 to 190. At the time of development, in Kim’s study [44], the Cronbach’s α of the tool was 0.925 for the intimacy and rationality factor, 0.829 for the control factor, 0.794 for the overprotection factor, and 0.645 for the neglect factor. The overall Cronbach’s α in this study was 0.921.

#### 2.3.2. Attitudes toward Aggression Regulation Behavior

This study used the tool for attitudes toward aggression regulation behavior from the aggression regulation behavior measurement tool developed by Jang and Ahn [20]. This tool was based on the early adolescent aggression tool of Ha and Kim [45], the peer conflict scale (PCS) produced by Marsee et al. [46], and Jang and Ahn’s [34] aggression measurement tool [19]. This tool includes two subfactors (evaluation attitudes and acceptance attitudes) and consists of six items in total: four items plus two additional items for each type. The item format is a 5-point semantic scale, and the scores ranged from 6 to 30 points. A higher score means a positive attitude toward the aggression regulation behavior. For construct validity, an exploratory factor analysis was performed, and at the time of development, in Jang and Ahn’s study [34], the Cronbach’s α of the entire tool was 0.894, whereas the overall tool’s Cronbach’s α in this study was 0.914. 

#### 2.3.3. Subjective Norms for Aggression Regulation Behavior

This study used the subjective norm tool for aggression regulation behavior from the aggression regulation behavior measurement tool developed by Jang and Ahn [20]. This tool was based on the early adolescent aggression tool of Ha and Kim [45], the peer conflict scale (PCS) produced by Marsee et al. [46], and Jang and Ahn’s [34] aggression measurement tool based on Ajzen’s TPB [19]. This tool includes two subfactors, namely, the overt norm and the relational norm, which have a total of six items on a 5-point Likert scale. The score ranges from a minimum of 6 to a maximum of 30, with higher scores indicating higher subjective norms. This means that the higher the measurement score, the greater the pressure from other people regarding the aggression regulation behavior. An exploratory factor analysis was performed for construct validity, in Jang and Ahn’s study [34], and the Cronbach’s α of the tool at the time of development was 0.886. The Cronbach’s α in this study was 0.891.

#### 2.3.4. Perceived Behavioral Control for Aggression Regulation Behavior

This study used the perceived behavioral control tool for aggression regulation behavior from the aggression regulation behavior measurement tool developed by Jang and Ahn [20]. This tool was based on the early adolescent aggression tool of Ha and Kim [45], the peer conflict scale (PCS) produced by Marsee et al. [46], and Jang and Ahn’s [34] aggression measurement tool based on Ajzen’s TPB [19]. This tool includes two subfactors, namely, overt control and relational control, and there is a total of six items on a 5-point Likert scale. The score ranges from a minimum of 6 points to a maximum of 30 points, and the higher the score, the higher the perceived behavioral control in terms of the aggression regulation behavior. That is, the higher the score, the better the middle school students could control external factors such as the person or situation they wanted to attack. For construct validity, an exploratory factor analysis was performed, and at the time of development, in Jang and Ahn’s study [34], the Cronbach’s α of the tool was 0.887. The Cronbach’s α in this study was 0.885.

#### 2.3.5. Difficulties in Emotion Regulation

This study used the scale for difficulties in emotion regulation (DERS) for Korean adolescents developed by Park [47], which is based on the emotion regulation difficulty scale developed by Gratz and Roemer [14], but which Park made easier to understand and adapted for adolescents. The DERS for adolescents includes six subfactors, such as impulse control difficulties, lack of emotional awareness, nonacceptance of emotional responses, lack of emotional clarity, limited access to emotional regulation strategies, and difficulties engaging in goal-directed behavior, and consists of a total of 34 items. The item format is a 5-point Likert scale, and a higher score indicates a higher degree of emotion regulation difficulty. In Park’s study [47], the total Cronbach’s α was 0.926, and the total Cronbach’s α in this study was 0.910.

#### 2.3.6. Aggression Regulation Intention

This study used the aggression regulation intention tool for aggression regulation behavior from the aggression regulation behavior measurement tool developed by Jang and Ahn [20]. This tool was based on the early adolescent aggression tool of Ha and Kim [45], the peer conflict scale (PCS) produced by Marsee et al. [46], and Jang and Ahn’s [34] aggression measurement tool based on Ajzen’s TPB [19]. This tool is divided into two subdomains, namely, overt aggression regulation intention and relational aggression regulation intention, and there are four items and two items for each area, respectively, for a total of six items. The item format is a 5-point Likert scale (1 = strongly disagree, 2 = somewhat disagree, 3 = moderate, 4 = slightly agree, and 5 = strongly agree), with the score ranging from 6 to 30. This means that the higher the score, the higher the aggression regulation intention. For construct validity, an exploratory factor analysis was performed, in Jang and Ahn’s study [34], and at the time of development, the Cronbach’s α of the tool was 0.894, whereas the Cronbach’s α in this study was 0.898.

#### 2.3.7. Aggression

This study used the aggression measurement scale (Korean-PCS) that Ha and Kim [45] modified, supplemented, and validated to suit the understanding of early middle school students, and which was developed from the peer conflict scale (PCS) produced by Marsee et al. [46]. The K-PCS includes four subtypes including proactive overt aggression, reactive overt aggression, proactive relational aggression, and reactive relational aggression, and it consists of 16 items in total, with four items for each type. The item format is a 5-point Likert scale (1 = strongly disagree, 2 = somewhat disagree, 3 = moderate, 4 = somewhat agree, and 5 = strongly agree), with a score ranging from 16 to 80 points. The higher the overall score, the greater the degree of aggression. The overall Cronbach’s α was 0.90 in Ha and Kim [45] and the Cronbach’s α for each factor was also calculated: that of proactive overt aggression was 0.74, that of reactive overt aggression was 0.77, that of proactive relational aggression was 0.72, and that of reactive relational aggression was 0.76. The overall Cronbach’s α in this study was 0.967.

### 2.4. Data Collection Methods and Ethical Considerations

The data for this study were collected from middle school students from two schools by using a structured self-report questionnaire. The data collection lasted for about 2 months, from July to September 2016, after IRB approval. For the sampling method, a convenience sample of middle schools in city D was used. After asking the homeroom teachers and subjects for cooperation by explaining the purpose of the study and how to respond to the questionnaire, as well as guaranteeing their anonymity, the questionnaire was distributed. We asked the subjects to first read the consent form in the classroom to understand the purpose of the study and to provide an anonymous signature to give consent to participate. Students under the age of 14 needed to obtain the signature of their guardian, in consideration of ethical aspects. Since the study considered the sensitive emotional issue of aggression, anonymity and confidentiality were essential to ensure the quality of the data. Therefore, all questionnaires were delivered in opaque envelopes, and after the questionnaires had been filled out, they were sealed and collected. 

For the protection of subjects, this study was reviewed by E University’s Institutional Bioethics Review Committee and received research approval (No. EU16-02) on 10 March 2016. The questionnaires were filled out by the subjects themselves, and a small gift was provided. To prevent the leakage of personal information, the researcher who analyzed the collected questionnaires coded subjects using numbers instead of names.

### 2.5. Data Analysis Method

The collected data were analyzed using SPSS WIN 26.0 and AMOS 26.0. Descriptive statistics were used as the general characteristics and research variables of the subjects. To examine the relevance of the study variables, the correlations were analyzed with Pearson’s correlation coefficient. The reliability of the internal consistency of the measurement tools was analyzed via Cronbach’s α. Exploratory factor analysis was used for the validity of measurement tools, and principal component analysis and varimax method were performed. To confirm the convergence and discriminant validity of the measurement tool, a confirmatory factor analysis was performed, and a covariate structural analysis was performed using the maximum likelihood method to test the fit of the model and test the hypothesis. For the fit test of the hypothetical model, the absolute fit measures of χ^2^ and df, the goodness-of-fit index (GFI), the adjusted goodness-of-fit index (AGFI), the double root of the root mean square error of approximation (RMSEA), and the standardized root mean square residual (SRMR) were obtained. The comparative fit index (CFI) and the incremental fit index (IFI) were used as incremental fit measures. As parameter estimates of the measurement model, the standardized regression weight (SRW), regression weight (RW), critical ratio (CR), and squared multiple correlations (SMCs) were used for analysis. The bootstrapping method was used to verify the statistical significance of the indirect and total effects of the model.

## 3. Results

### 3.1. Subject Characteristics

In total, 310 subjects were surveyed, of which there were 241 (77.7%) first-year students and 69 (22.3%) second-year students. There were 116 male students (37.4%) and 194 female students (62.6%). Regarding religion, 163 people were nonreligious (52.6%), which was the largest group. For the father’s occupation, there were 95 office workers (30.6%), and for the mother’s occupation, there were 129 housewives (24.5%). Regarding the economic status of households, 189 (61.0%) subjects recognized that they were middle class (Table 1).

### 3.2. Descriptive Statistics and Confirmatory Factor Analysis of the Research Variables

If we consider the average of the measured variables, the scores for positive parenting behavior within the parenting behaviors was 3.67 ± 0.70 and that of negative parenting behavior was 2.55 ± 0.64 points. Among the attitudes toward aggression regulation behavior, the score of the evaluation attitude was 2.91 ± 1.0 and that of acceptance attitude was 3.18 ± 0.97. Among the subjective norms, the overt norms scored 4.14 ± 0.82 and the relational norms scored 4.33 ± 0.82. Within perceived behavioral control, overt control scored 3.74 ± 0.90 and relational control scored 3.79 ± 1.0. Among the difficulties in regulating emotion, impulse control difficulty scored 2.71 ± 0.78 points, emotion insensitivity scored 2.74 ± 0.90 points, lack of emotional clarity scored 2.69 ± 0.94 points, restricted access to emotion regulation strategies scored 2.75 ± 0.83 points, and difficulty in performing goal-oriented behavior scored 3.19 ± 0.77 points. For aggression regulation intention, the score of overt aggression regulation intention was 3.75 ± 0.90 and that of relational aggression regulation intention was 3.73 ± 1.0. For aggression, proactive overt aggression scored 1.62 ± 0.81 points, reactive overt aggression scored 1.76 ± 0.91 points, proactive relational aggression scored 1.63 ± 0.82 points, and reactive relational aggression scored 1.75 ± 0.90 points (Table 2).

**Figure 2:**
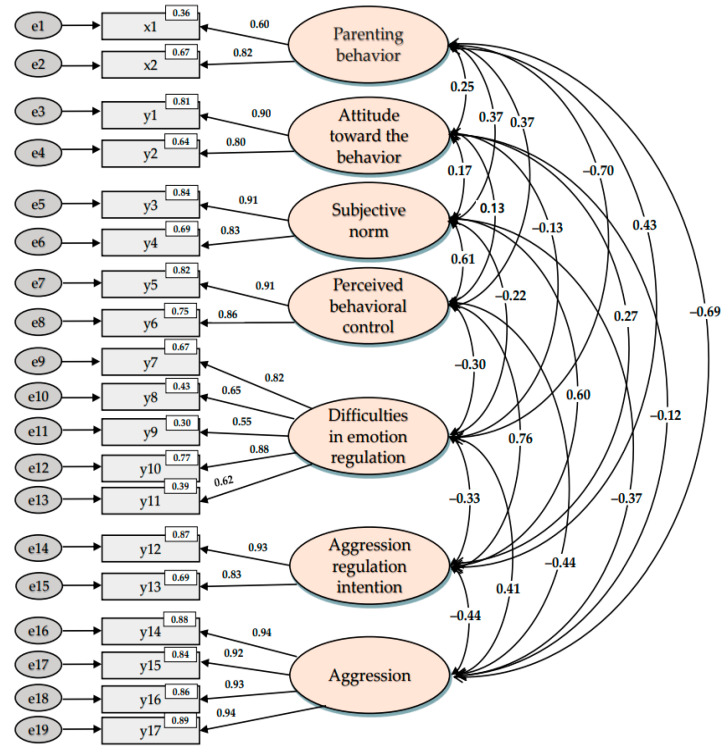

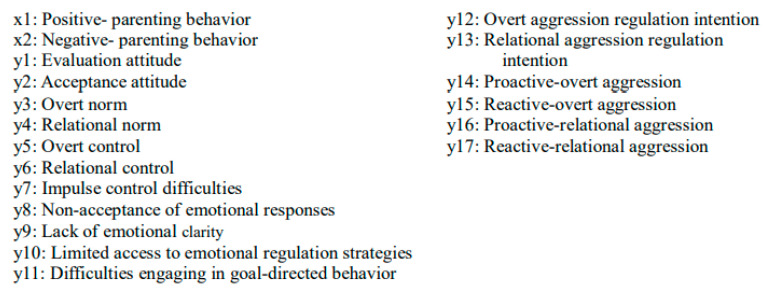
Confirmatory factor analysis.

Since the structural equation model is an analysis performed under the assumption of multivariate normality, normality was verified by confirming whether the study variables satisfied the assumption of normality. A case where the skewness is less than the absolute value of 2 and the kurtosis is less than the absolute value of 4 is regarded as an assumption of univariate normality [40]. In this study, the skewness of the measurement variables was between −1.187 and 1.271, and the kurtosis was between −0.844 and 1.413, satisfying the assumptions of a univariate normal distribution. As a result of the confirmatory factor analysis, the difficulties in emotion regulation subfactor “lack of attention and awareness of emotion” was removed because the standardized regression weight was 0.231, which did not reach the 0.5 level [40]. As a result of reconducting the confirmatory factor analysis, the correlation coefficients ranged from 0.121 to 0.764 in absolute values, and all distributions were less than 0.90; thus, the problem of multicollinearity did not appear and was excluded (Figure 2).

The standardized regression coefficients ranged from 0.549 to 0.942, all of which were above 0.50; the average variance extracted (AVE) value of the study variables was 0.5 or more and the construct reliability (CR) value was 0.7 or more, confirming the convergent validity (Table 3).

It was also found that the average variance extracted (AVE) value was larger than the square of the correlation coefficients among the latent variables (Ø^2^). Discriminant validity was confirmed because the confidence interval r ± 2 × SE of the correlation coefficient did not include 1 (Table 4).

### 3.3. Verification of the Structural Model of Middle School Students’ Aggression

#### 3.3.1. Validation of the Hypothetical Model

The hypothetical model that the TPB applied to aggression was affected by parenting behavior and that aggression was affected by difficulties in emotion regulation was verified through a fitness test in accordance with the actual data. If the model fit index is greater than 0.90 or the RMSEA is less than 0.05, the model fit is excellent. If the model fit index is between 0.80 and 0.90, or the RMESA has a value between 0.05 and 0.08, the model fit is judged to be relatively good [40]. From the results of testing the fit of the hypothetical model in this study, where Χ^2^ = 359.984 (*p* < 0.001), DF =138, CMIN/DF =2.609, GFI = 0.885, AGFI = 0.841, IFI = 0.946, CFI = 0.946, SRMR = 0.069, and RMSEA = 0.072, it was found that the model reached a relatively acceptable level (Table 5).

**Figure 3:**
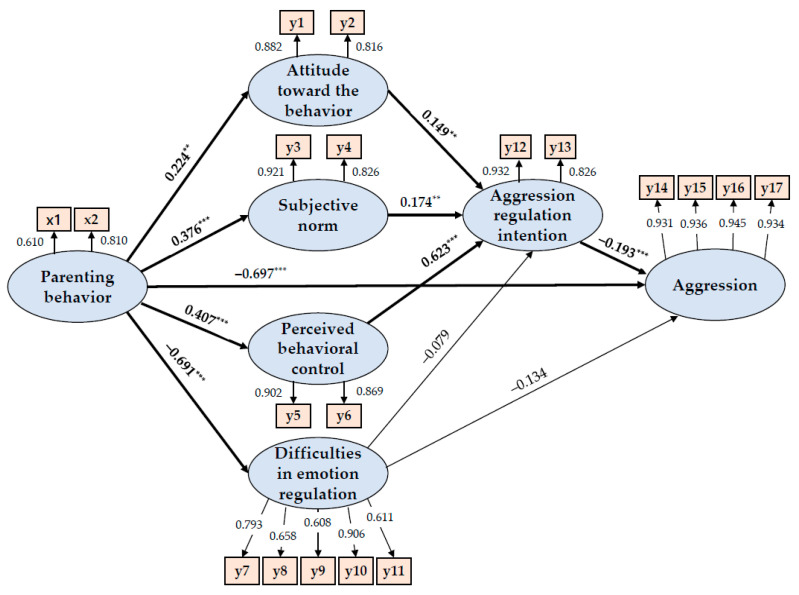

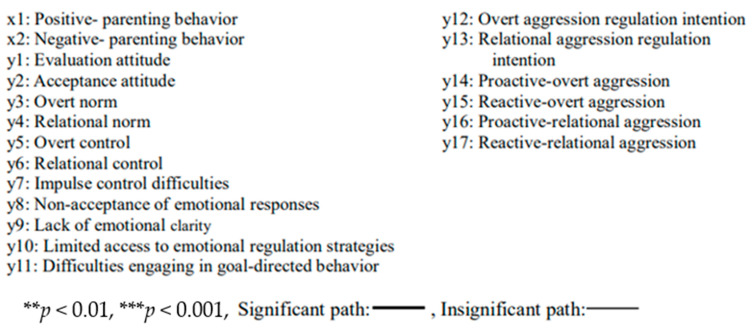
Path diagram of the hypothetical model.

Therefore, the hypothetical model was confirmed as the final model without modifications (Figure 3).

#### 3.3.2. Estimating the Parameters of the Model and Analyzing the Effects

As the results of the analysis of the hypothetical model of this study showed, 9 out of 11 pathways were statistically significant, and 2 were not significant. Parenting behavior had a significant, direct effect on attitudes toward aggression regulation behavior (γ = 0.224, *p* = 0.002), subjective norms (γ = 0.376, *p* < 0.001), perceived behavioral control (γ = 0.407, *p* < 0.001), and difficulty in regulating emotion (γ = −0.691, *p* < 0.001). Perceived behavioral control (β = 0.623, *p* < 0.001), subjective norms (β = 0.174, *p* = 0.005), and attitudes toward aggression regulation behavior (β = 0.149, *p* = 0.002) had significant, direct effects on aggression regulation intention. Difficulties in emotion regulation (β = −0.079, *p* = 0.092) had no significant, direct effect on aggression regulation intention. The explanatory power of these variables for aggression regulation intention was 64.0%. In the final results of this study, parenting behavior had a significant, direct effect on lowering the aggression of middle school students (β = −0.697, *p* < 0.001), but the indirect effect was not significant. Difficulties in emotion regulation had no significant, direct effect (β = −0.134, *p* = 0.148) or indirect effect (β = 0.015, *p* = *0*.097) on aggression. Aggression regulation intention (β = −0.193, *p* < 0.001) had a significant, direct effect on lowering aggression (Table 6). These variables had an explanatory power for aggression of 50.5% (Table 7).

## 4. Discussion

This study applied Ajzen’s TPB [19] to explain aggression in middle school students. In addition, a hypothetical model was constructed and the significance of the model was verified by adding variables regarding parenting behavior and emotion regulation that affect aggression. The variables of the TPB that were applied to explain aggression were attitudes toward aggression regulation behavior, subjective norms, perceived behavioral control, and aggression regulation intention. In this study, the structural model of aggression in middle school students showed an acceptable level of model fit, and it was judged to be a suitable model to explain aggression in middle school students.

### 4.1. Parenting Behavior and Difficulties in Emotion Regulation

The biggest factor influencing aggression in middle school students was parenting behavior. In other words, it was found that aggression in middle school students is greatly affected by parenting behavior. Looking at the correlation between parenting behavior and aggression, there was a statistically significant negative correlation (r = −0.560). As intimacy and reasonable positive parenting behavior increased, aggression decreased, which confirmed that when the level of controlling, overprotective, and neglectful negative parenting behavior was higher, aggression in middle school students was also higher. These results are in line with a study that showed that adolescents’ aggression levels are lowered when parenting behavior is positive [13]. Parenting behavior is an important factor influencing adolescent development. In particular, the home is the basic environment where adolescents observe the behavior of their parents, and parents greatly influence their children’s emotional and behavioral development through interactions with their children [48]. In this respect, parent education programs on desirable parenting behavior support the fact that aggression in middle school students can be prevented. Therefore, a method to promote positive parenting behavior in parents is needed.

In this study, difficulties in emotion regulation did not show any direct or indirect effects on aggression regulation intention or aggression. These results are different from those of a study showing that adolescents’ emotion regulation factors affect the tendency toward aggression and that the negative parenting behavior of fathers affects the children’s aggression by mediating the children’s difficulties in emotion regulation [49,50]. To understand the results of these studies, first, we looked at the correlation between difficulties in emotion regulation and aggression regulation intention in this study, and found that overt aggression regulation intention and relational aggression regulation intention were not significantly correlated with the unacceptability of emotion. In Park’s study [47] that validated the tools for measuring difficulties in emotion regulation for Korean adolescents, the item “When I feel bad, I start to evaluate myself negatively,” was not related to accessing an effective emotion regulation strategy, but was associated with the difficulty of accepting felt emotions. Because our culture is not very receptive to the feeling of anger, participants said that it was not right to be angry and that they did not want to acknowledge their negative emotions. Thus, it can be seen that there was a discrepancy between the negative meaning of difficulties in emotion regulation and the positive meaning of aggression regulation intention experienced. In addition, the subjects in this study had a high level of perceived positive parenting behavior, and the average score of aggression was 1.69, which was relatively low. In previous studies, when the parenting behavior, which is an environmental factor, was negative, it was reported that this could lead to aggression in children through changes in emotion regulation, which is the child’s internal mechanism [50]. Conversely, in this study, the perceived level of positive parenting behavior was high, and thus, it is thought that difficulties in emotion regulation did not affect aggression. In other words, it was shown that positive parenting behavior can lead to a decrease in aggression without causing difficulties in regulating emotion in middle school students. In conclusion, this study recognized that parenting behavior is the most important factor affecting aggression in middle school students, and showed that education to improve positive parenting behavior should be carried out to prevent or reduce aggression in middle school students.

### 4.2. Aggression Regulation Intention and Aggression Based on the TPB

The TPB is the theory that attitudes toward behavior, subjective norms, and perceived behavioral control influence behavior intention, and that behavior intentions can explain behavior. To explain aggression, this study analyzed attitudes toward aggression regulation behavior, subjective norms, perceived behavioral control, and aggression regulation intention. Based on this, the results of this study are described below.

First, attitudes toward aggression regulation behavior, subjective norms, and perceived behavioral control influenced aggression regulation intention. Moreover, aggression regulation intention was confirmed to have a significant effect on aggression. This was consistent with the conceptual framework of the TPB. Studies that have explained aggression by applying the TPB include a study explaining aggression in university athletes and a study on the effect of violent PC game experiences, aggressive characteristics, and self-efficacy regarding aggression intentions and aggressive behavior in university students [28,51]. In particular, the study explaining aggression in university athletes explained the aggression by suggesting the effect of exercise behavior intention on aggression. The attitudes toward exercise and perceived behavioral control of university athletes had an effect on exercise behavior intention, and exercise behavior intention had an effect on aggression [28]. This study was similar to our study in that it tried to explain the concept of aggression by applying the TPB to explain that aggression is reduced through intention. However, this study explained that aggression was mediated by the concept of aggression regulation intention, which can lower aggression, whereas the previous study was different from this study in that it claimed that aggression was mediated by the concept of exercise behavior intention. Moreover, the subjective norms of university athletes did not affect exercise behavior intention. In this study, the subjective norms of middle school students were found to affect aggression regulation intention, and the results of the previous study were different from the results of this study. The reason seems to be the difference in the perception of pressure from significant people around the study subjects. That is, it was judged that middle school students are under more social pressure than university students. Accordingly, it was confirmed that the age of the study subjects affects the relationships of the variables affecting aggression to which the TPB is applied.

Second, in this study, perceived behavioral control had a significant positive direct effect and was the variable that had the greatest influence on aggression regulation intention. In a meta-analysis of physical activity research applying the TPB, it was reported that the TPB’s components of attitudes toward behavior, subjective norms, and perceived behavioral control have a consistent effect on behavior intention, but perceived behavioral control had a greater effect on behavior intention than attitudes toward a behavior [52]. In addition, in a study on the effects of violent PC game experiences, aggressive characteristics, and self-efficacy regarding aggression intention and behavior in university students, it was reported that self-efficacy, a concept similar to perceived behavioral control, had the greatest explanatory power for aggression intention [51]. In this study, perceived behavioral control was found to be more influenced by parenting behavior than by other variables. As a result, it is thought that perceived behavioral control has a higher effect on aggression regulation intention than other variables. In other words, this finding shows that the ability to better control oneself (perceived behavioral control) in situations that can become aggressive can increase the intention to regulate aggression. However, in a study explaining aggression in university athletes, it was reported that attitudes toward exercise had the highest effect on exercise behavior intention rather than perceived behavioral control [28]. Moreover, in a study on the survival, brain death, and organ donation intentions of university nursing students, subjective norms had the greatest effect on behavior intention [53]. These various results show that although behavior intention is determined by attitudes toward behavior, subjective norms, and perceived behavioral control, the influence of these factors depends on differences in the sex, age, and cultural characteristics of the study subjects [54]. Because the subjects of this study were middle school students, it was concluded that perceived behavioral control was highly influenced by parenting behavior, and thus, this variable increased aggression regulation intention. As a result, it can be said that middle school students are still very much affected by parenting behavior. In addition, the results of this study show that perceived behavioral control has a strong, direct effect on aggression regulation intention, indicating that perceived behavioral control should be enhanced in order to improve aggression regulation intention in middle school students, which could ultimately reduce aggression in middle school students.

Third, aggression regulation intention was a major influencing factor that explained aggression. In this study, TPB was applied to the method of expression that classified aggression into a purpose-oriented form (overt) and a non-purpose-oriented form (relational) [48]. Accordingly, aggression regulation intention decreased all factors of proactive overt aggression, reactive overt aggression, proactive relational aggression, and reactive relational aggression. In addition, previous studies that applied the TPB classified aggression into behavioral aspects (physical and verbal), emotional aspects (anger), and cognitive aspects (hostility) [55]. As a result, exercise behavior intention did not reduce verbal aggression in terms of behavioral aspects, but decreased anger and hostility in terms of emotional and cognitive aspects, and physical aggression factors in terms of behavioral aspects [28]. Therefore, in the application of the TPB to aggression, it was confirmed that behavior intention affects aggression. These results reported that behavior intention is the antecedent factor of behavior, and the basic assumption of the theory is that attitudes, subjective norms, and perceived behavioral control affect behavior intention, supporting the results of a meta-analysis of the usefulness of the social cognitive behavior model [56]. However, the effects of aggression regulation intention on the subfactors of aggression in this study ranged from -0.179 to -0.182. The effect of exercise behavior intention in previous studies on the subfactor of aggression was 0.048–0.282. In both studies, the effects were not relatively high [28]. In addition, in a study on the relationship between violent PC game experiences and aggressive behavior, aggression intention did not have a significant effect on aggression [51]. This indicates that the ability of behavior intention is limited in sufficiently explaining aggression as influenced by attitudes toward the behavior, the subjective norms, and perceived behavioral control. To compensate for this, it is not enough to regulate aggression only through educational strategies that are meant to improve desirable aggression regulation intentions. Moreover, the fact that exercise behavior intention did not affect verbal aggression showed that it is necessary to allow middle school students’ aggression to be released in an appropriate direction [28], and thus, aggression can be reduced by using educational strategies that allow them to fully express their feelings so that they can understand and persuade others and express their assertions through nonaggressive words. In addition, in this study, the effect of parenting behavior on aggression was higher than the effect on aggression regulation intention, which can be interpreted as showing that it is difficult to explain aggression in middle school students by aggression regulation intention alone. Therefore, in addition to aggression regulation intention, research into other major variables that can explain aggression should be conducted.

To summarize this discussion, attitudes toward aggression regulation behavior, subjective norms, and perceived behavioral control influenced aggression regulation intention, and aggression regulation intention had an effect on reducing aggression. Therefore, the results of this study were consistent with the conceptual framework of the TPB. In addition, when variables of parenting behavior and difficulties in emotion regulation were added to the TPB, parenting behavior was the factor that had the strongest influence on aggression in middle school students, and it further enhanced the explanatory power of the factors influencing aggression. Thus, this study’s extended aggression explanatory model for reducing aggression adequately explained aggression in middle school students.

### 4.3. Research Implications

#### 4.3.1. Theoretical Aspects

This study analyzed the aggression of middle school students by verifying the causal relationship between factors that can affect aggression in middle school students and by selecting variables of parenting behavior and emotional regulation difficulties based on Ajzen’s (1991) TPB and a review of previous studies. The significance of this study is that it has built a hypothetical model that can provide a theoretical basis for related research in the future.

#### 4.3.2. Research Aspects

This study is meaningful in that it confirmed the significance of specific pathways by identifying the direct and indirect effects of each factor influencing aggression by injecting parental behavior and emotional dysregulation variables into a hypothetical model. In addition, it is meaningful in that it can be the basis for a study to develop an aggression control training program for middle school students based on the variables of this study and can be used to evaluate the program’s effectiveness.

#### 4.3.3. Estimating the Parameters of the Model and Analyzing the Effects

This study sought ways to reduce aggression by identifying the influencing factors on middle school students’ aggression. It is thought that an aggression control training program for reducing aggression in middle school students will contribute to maximizing the control of aggression when it is applied as part of community and youth mental health projects.

### 4.4. Limitations of the Research

This study established and verified a research model for middle school students’ aggression, and has the following limitations: (1) The subjects of this study are first and second graders in middle school in Korea, and it is necessary to be careful in generalizing the results to other adolescents. (2) This study relies on a subjective evaluation since the research subjects are surveyed through a self-administered questionnaire. (3) Since this study validated the model with data collected cross-sectionally, there may be limitations in comprehensively explaining aggression. This is due to the lack of measurement tools that can clearly verify and reflect each essential concept. Therefore, tool development, longitudinal studies, and repeated studies are additionally needed to study various age groups.

## 5. Conclusions

### 5.1. Conclusions

This study verified the relationship between the influencing factors that explain aggression based on the TPB. This study found that middle school students are greatly influenced by parenting behavior and are under high social pressure due to their specific age group. Thus, to reduce and regulate aggression in middle school students, parents should adopt desirable parenting behaviors. According to the results of the TPB applied to aggression, middle school students are required to have positive attitudes toward aggression regulation behavior, high subjective norms, high perceived behavioral control, and high aggression regulation intention. In particular, perceived behavioral control had a strong effect on aggression regulation intention. Therefore, to increase aggression regulation intention, a plan should be developed to improve the ability to control oneself well (perceived behavioral control) in situations that can become aggressive. In conclusion, according to this study, the best way to increase aggression regulation intention is to improve perceived behavioral control, and various methods can be used to reduce aggression, including desirable parenting behavior, a positive attitude toward aggression regulation behavior, high subjective norms, high perceived behavioral control, and high aggression regulation intention.

### 5.2. Suggestions

Based on this study’s results, we suggest the following: (1) using the model of this study, we suggest applying various strategic behavioral intentions to reduce aggression. (2) Although this study is only related to middle school students’ aggression, we suggest research or model building research including concepts not mentioned in this study. (3) We also suggest longitudinal or qualitative research to clarify the before and after relationships of variables that are difficult to identify through cross-sectional studies. (4) Based on the results of this study, we suggest developing a nursing intervention program to improve middle school students’ ability to control aggression. (5) Finally, we suggest adding another variable that can explain aggression by applying a model other than the TPB.

## Figures and Tables

**Table 1:** Demographic characteristics of the participants.

(*N =* 310)			
Characteristics	Categories	n (%)	
Grade	Middle school 1	241	(77.7)
	Middle school 2	69	(22.3)
Gender	Male	116	(37.4)
	Female	194	(62.6)
Religion	None	163	(52.6)
	Christianity	98	(31.6)
	Catholic	23	(7.4)
	Buddhism	21	(6.8)
	Other	5	(1.6)
Father’s job type	None (or house wife)	40	(12.9)
	Office job	26	(8.4)
	Employee	95	(30.6)
	Professional position	36	(11.6)
	Seller, service	36	(11.6)
	Personal business	55	(17.8)
	Other	22	(7.1)
Mother’s job type	None (or house wife)	129	(41.6)
	Office job	14	(4.5)
	Employee	51	(16.5)
	Professional position	23	(7.4)
	Seller, service	53	(17.1)
	Personal business	21	(6.8)
	Other	19	(6.1)
Family income	High	85	(27.4)
	Average	189	(61.0)
	Low	36	(11.6)

**Table 2:** Descriptive statistics of the measured variables.

Latent Variables Measured Variables	Item No	Min	Max	M ± SD	Skewness	Kurtosis
Parenting behavior	38	2	4.53	3.11 ± 0.34	0.526	1.417
Positive parenting behavior	19	1.42	5	3.67 ± 0.70	−0.106	−0.594
Negative parenting behavior	19	1.16	4.58	2.55 ± 0.64	0.174	−0.281
Attitude toward the behavior	6	1	5	2.95 ± 0.98	−0.019	−0.305
Evaluation attitude	4	1	5	2.91 ± 1.0	0.045	−0.531
Acceptance attitude	2	1	5	3.18 ± 0.97	−0.201	−0.082
Subjective norm	6	1	5	4.08 ± 0.76	−0.719	0.308
Overt norm	4	1	5	4.14 ± 0.82	−0.985	0.632
Relational norm	2	1	5	4.33 ± 0.82	−1.187	0.933
Perceived behavioral control	6	1	5	3.76 ± 0.84	−0.395	−0.176
Overt control	4	1	5	3.74 ± 0.90	−0.407	−0.395
Relational control	2	1	5	3.79 ± 1.0	−0.454	−0.611
Difficulties in emotion regulation	34	1.15	3.94	2.67 ± 0.56	−0.463	−0.375
Impulse control difficulties	6	1	5	2.71 ± 0.78	−0.156	−0.2
Lack of emotional awareness	7	1	3.86	2.20 ± 0.66	−0.021	−0.918
Non-acceptance of emotional responses	7	1	5	2.74 ± 0.90	−0.038	−0.519
Lack of emotional clarity	3	1	5	2.69 ± 0.94	0.114	−0.507
Limited access to emotional regulation strategies	7	1	5	2.75 ± 0.83	−0.105	−0.302
Difficulties engaging in goal-directed behavior	4	1	5	3.19 ± 0.77	−0.435	0.344
Aggression regulation intention	6	1	5	3.75 ± 0.89	−0.412	−0.303
Overt aggression regulation intention	4	1	5	3.75 ± 0.90	−0.353	−0.484
Relational aggression regulation intention	2	1	5	3.73 ± 1.0	−0.455	−0.392
Aggression	16	1	4.25	1.69 ± 0.81	1.066	−0.032
Proactive-overt aggression	4	1	4.25	1.62 ± 0.81	1.168	0.233
Reactive-overt aggression	4	1	4.75	1.76 ± 0.91	1.039	0.014
Proactive-relational aggression	4	1	4.75	1.63 ± 0.82	1.271	0.560
Reactive-relational aggression	4	1	4.25	1.75 ± 0.90	0.959	−0.325

**Table 3:** Parameter estimates of confirmatory factor analysis, AVE and CR.

Latent Variables Measured Variables	RW	SRW	S.E.	C.R	*p*	AVE	CR
Parenting behavior						0.694	0.816
Positive parenting behavior	1.000	0.602					
Negative parenting behavior	1.252	0.816	0.131	9.579	<0.0001		
Attitude toward the behavior						0.721	0.837
Evaluation attitude	1.000	0.897					
Acceptance attitude	0.811	0.802	0.135	5.991	<0.001		
Subjective norm						0.827	0.905
Overt norm	1.000	0.915					
Relational norm	0.899	0.831	0.064	13.946	<0.001		
Perceived behavioral control						0.793	0.885
Overt control	1.000	0.907					
Relational control	1.075	0.865	0.059	18.181	<0.001		
Difficulties in emotion regulation						0.585	0.872
Impulse control difficulties	1.000	0.819					
Non-acceptance of emotional responses	0.926	0.652	0.078	11.894	<0.001		
Lack of emotional clarity	0.810	0.549	0.083	9.710	<0.001		
Limited access to emotional regulation strategies	1.147	0.877	0.069	16.668	<0.001		
Difficulties engaging in goal- directed behavior	0.753	0.625	0.067	11.291	<0.001		
Aggression regulation intention						0.785	0.879
Overt aggression regulation intention	1.000	0.931					
Relational aggression regulation intention	0.984	0.828	0.057	17.410	<0.001		
Aggression						0.897	0.972
Proactive-overt aggression	1.000	0.937					
Reactive-overt aggression	1.100	0.917	0.037	29.776	<0.001		
Proactive-relational aggression	1.010	0.928	0.033	30.965	<0.001		
Reactive-relational aggression	1.116	0.942	0.034	32.857	<0.001		

RW = regression weight; SRW = standardized regression weight; SE = standard error; C.R. = critical ratio; AVE = average variance extracted; CR = construct reliability.

**Table 4:** Correlations among the latent variables.

Variables	X1	Y1	Y2	Y3	Y4	Y5	Y6
X1	1						
Y1	0.189 **	1					
Y2	0.356 ***	0.148 **	1				
Y3	0.363 ***	0.115 *	0.531 ***	1			
Y4	−0.500 ***	−0.088	−0.151 **	−0.199 ***	1		
Y5	−0.405 ***	0.231 ***	0.513 ***	0.677 ***	−0.234 ***	1	
Y6	−0.560 ***	−0.118*	−0.345 ***	−0.397 ***	0.359 ***	−0.407 ***	1

X1 = Parenting behavior; Y1 = attitude toward the behavior; Y2 = subjective norm; Y3 = perceived behavioral control; Y4 = difficulties in emotion regulation; Y5 = aggression regulation intention; Y6= aggression. * *p* < 0.05, ** *p* < 0.01, *** *p* < 0.01.

**Table 5:** Model fit of the confirmatory factor analysis and the hypothetical model.

Model	CMIN(Χ^2^)			CMIN/DF	GFI	AGFI	IFI	CFI	SRMR	RMSEA
	Χ^2^	DF	*p*							
Excellent			≥0.05	≤3	≥0.90	≥0.90	≥0.90	≥0.90	≤0.08	0~0.05
Above average			≥0.05	≤4	≥0.80	≥0.80	≥0.80	≥0.80	≤0.10	0.05~0.08
Confirmatory factor analysis	387.700	131	*p* < 0.001	2.960	0.876	0.820	0.938	0.937	0.067	0.080
Research model	359.984	138	*p* < 0.001	2.609	0.885	0.841	0.946	0.946	0.069	0.072

GFI—goodness-of-fit index; AGFI—adjusted goodness-of-fit index; IFI—incremental fit index; CFI—comparative fit index; SRMR—standardized root mean residual; RMSEA—root mean square error of approximation.

**Table 6:** Direct effects, indirect effects, and total effects of the hypothetical model.

Exogenous Variables		Endogenous Variables	Standardized Direct Effects β(*p*)	Standardized Indirect Effects β(*p*)	Standardized Total Effects β(*p*)
(H1)	Parenting behavior	Attitude toward the behavior	0.224 (0.002) **		0.224 (0.002) **
(H2)	Parenting behavior	Subjective norm	0.376 (<0.001) ***		0.376 (<0.001) ***
(H3)	Parenting behavior	Perceived behavioral control	0.407 (<0.001) ***		0.407 (<0.001) ***
(H4)	Parenting behavior	Difficulties in emotion regulation	−0.691 (<0.001) ***		−0.691 (<0.001) ***
(H5)	Attitude toward the behavior	Aggression regulation intention	0.149 (0.002) **		0.149 (0.002) **
(H6)	Subjective norm		0.174 (0.005) **		0.174 (0.005) **
(H7)	Perceived behavioral control		0.623 (<0.001) ***		0.623 (<0.001) ***
(H8)	Difficulties in emotion regulation		−0.079 (0.092)		−0.079(0.092)
(H9)	Parenting behavior	Aggression	−0.697 (<0.001) ***	0.014 (0.884)	−0.683 (0.004) **
(H10)	Difficulties in emotion regulation		−0.134 (0.148)	0.015 (0.097)	−0.118 (0.242)
(H11)	Aggression regulation intention		−0.193 (<0.001) ***		−0.193 (<0.001) ***

** *p* < 0.01, and *** *p* < 0.001.

**Table 7:** Parameter estimates of hypothetical model.

Exogenous Variables	Endogenous Variables	RW	SRW	S.E.	C.R.	*p*	SMC
Parenting behavior	Attitude toward the behavior	0.495	0.224	0.157	3.145	0.002 **	0.05
Parenting behavior	Subjective norm	0.669	0.376	0.127	5.258	<0.001 ***	0.142
Parenting behavior	Perceived behavioral control	0.776	0.407	0.139	5.579	<0.001 ***	0.165
Parenting behavior	Difficulties in emotion regulation	−0.996	−0.691	0.126	−7.917	<0.001 ***	0.477
Attitude toward the behavior	Aggression regulation intention	0.133	0.149	0.043	3.054	0.002 **	0.64
Subjective norm		0.192	0.174	0.068	2.82	0.005 **	
Perceived behavioral control		0.642	0.623	0.068	9.484	<0.001 ***	
Difficulties in emotion regulation		−0.107	−0.079	0.064	−1.686	0.092	
Parenting behavior	Aggression	−1.225	−0.697	0.213	−5.762	<0.001 ***	0.505
Difficulties in emotion regulation		−0.163	−0.134	0.112	−1.448	0.148	
Aggression regulation intention		−0.172	−0.193	0.051	−3.398	<0.001 ***	

RW= regression weight; SRW= standardized regression weight; SE= standard error; C.R.= critical ratio; SMC= squared multiple correlations. ** *p* < 0.01, *** *p* < 0.001.

## Data Availability

All data sets are available from the corresponding author by reasonable request.
